# Long-term genomic selection for heterosis without dominance in multiplicative traits: case study of bunch production in oil palm

**DOI:** 10.1186/s12864-015-1866-9

**Published:** 2015-08-29

**Authors:** David Cros, Marie Denis, Jean-Marc Bouvet, Leopoldo Sánchez

**Affiliations:** Genetic Improvement and Adaptation of Mediterranean and Tropical Plants Research Unit (UMR AGAP), CIRAD, 34398 Montpellier, France; Forest Tree Improvement, Genetics and Physiology Research Unit (UR AGPF), INRA, 45075 Orleans, France

**Keywords:** Genomic selection, GBLUP, Oil palm, Hybrid, Reciprocal recurrent selection

## Abstract

**Background:**

To study the potential of genomic selection for heterosis resulting from multiplicative interactions between additive and antagonistic components, we focused on oil palm, where bunch production is the product of bunch weight and bunch number. We simulated two realistic breeding populations and compared current reciprocal recurrent selection (RRS) with reciprocal recurrent genomic selection (RRGS) over four generations. All breeding strategies aimed at selecting the best individuals in parental populations to increase bunch production in hybrids. For RRGS, we obtained the parental genomic estimated breeding values using GBLUP with hybrid phenotypes as data records and population specific allele models. We studied the effects of four RRGS parameters on selection response and genetic parameters: (1) the molecular data used to calibrate the GS model: in RRGS_PAR, we used parental genotypes and in RRGS_HYB we also used hybrid genotypes; (2) frequency of progeny tests (model calibration); (3) number of candidates and (4) number of genotyped hybrids in RRGS_HYB.

**Results:**

We concluded that RRGS could increase the annual selection response compared to RRS by decreasing the generation interval and by increasing the selection intensity. With 1700 genotyped hybrids, calibration every four generations and 300 candidates per generation and population, selection response of RRGS_HYB was 71.8 % higher than RRS. RRGS_PAR with calibration every two generations and 300 candidates was a relevant alternative, as a good compromise between the annual response, risk around the expected response, increased inbreeding and cost. RRGS required inbreeding management because of a higher annual increase in inbreeding than RRS.

**Conclusions:**

RRGS appeared as a valuable method to achieve a long-term increase in the performance for a trait showing heterosis due to the multiplicative interaction between additive and negatively correlated components, such as oil palm bunch production.

## Background

Genomic selection (GS) [1] is the state-of-the-art method of marker-assisted selection for complex traits. GS relies on dense genome-wide marker coverage to produce genomic estimated breeding values (GEBV) from a joint analysis of all markers. GEBV can be obtained using a realized additive relationship matrix computed from markers, in what is called the GBLUP method [2, 3]. The GS model is calibrated using individuals with known phenotypes and genotypes (training set) and predicts the GEBV of selection candidates. For phenotypically evaluated candidates, GS is of interest due to its ability to give GEBV with higher accuracy (*r*_*ÂA*_, the correlation between true and estimated breeding values) than the EBV traditionally obtained through expected additive relationships computed from the pedigree. GS also gives GEBV of selection candidates that were only genotyped, thus allowing selection without phenotypic evaluation. This reduces the length of the generation interval (*L*), especially if conventional breeding requires long progeny tests, and increases the selection intensity (*i*) when the cost of phenotyping is higher than the cost of genotyping. Consequently, the annual selection response, which is given by *r*_*ÂA*_ *× i × σ*_*a*_*/L* (with *σ*_*a*_ the additive standard deviation) [4, 5], can be higher with GS than with phenotypic selection.

GS can be used to increase the performance of interpopulation plant hybrids and crossbred animals. Kinghorn et al. [6] simulated a crossbreeding system where GS was applied to select among parental lines in order to increase heterosis in crossbred animals for a trait with dominance at QTL. The highest selection response was obtained in their study with reciprocal recurrent genomic selection (RRGS), which consisted of using phenotypes and gametotypes of crossbred individuals to estimate line-specific marker effects. Heterosis in a complex trait can also result from the multiplicative interaction between additive and negatively correlated components ([7, 8] p68-71]). For example, this can be the case for yield in crops as a product of fruit weight and number, or plant height as a product of internode number and length. In such cases, dominance at QTL is not necessary to explain heterosis in the multiplicative trait. As GS proved to be efficient for single additive traits in many studies, it could also be beneficial in the case of multiplicative interactions between complementary parental components. However, this potential benefit over conventional phenotypic selection has not been quantified so far. Oil palm is an interesting model for this purpose. In oil palm, bunch production is the product of bunch weight (BW) and bunch number (BN), two negatively correlated and mostly additive traits [9, 10]. Oil palm breeding currently relies on reciprocal recurrent selection (RRS) between two heterotic populations showing complementary characteristics for BW and BN. One of them is the Deli population of Asian provenance and the other is African, commonly known as the La Mé population (Côte d’Ivoire). Deli palms have small numbers of large bunches, while La Mé have large numbers of small bunches. This results in heterosis in the hybrids for bunch production, which is at least 25 % higher than in the parental populations [11].

The potential of GS in oil palm has been evaluated in two studies. One of these is an empirical work [12], where the accuracy of GS was estimated through a cross-validation approach. In this previous study, we implemented population-specific GS models with progeny-tested parents as training sets, using their deregressed EBV and genotypes. This approach required little genotyping effort, as the number of progeny-tested parents was reduced (<200 per population and generation). On the other hand, these small numbers lead to small training sets, consequently with a detrimental effect for GS accuracy. The small number of individuals available to train the GS model is a common problem for many species requiring costly and time-consuming field trial evaluations, particularly in perennial crops (e.g. around 0.5 ha per cross and 15 years of data record in oil palm). An alternative to enlarge the training set could be to also include hybrid individuals, to take advantage of the allelic segregation existing within hybrid crosses due to heterozygosity in parents. For this purpose, a GS analysis taking hybrid gametotypes and parental genotypes into account could be implemented, as in Kinghorn et al. [6]. Furthermore, in our previous empirical study, a cross-validation approach was applied in a single generation, whereas it would be more interesting to assess the potential of GS over the long term, taking not only the selection accuracy but also the generation interval and selection intensity into account. Computer simulation is useful for this, in particular for species with long breeding cycles and extensive field trials [13].

The second study on the potential of GS in oil palm involved simulation, used to assess the potential of the method over three generations [14]. The authors concluded that GS gave a higher annual selection response than phenotypic and marker-assisted selection. However, they made simplifying assumptions: considering a single additive trait instead of the multiple trait approach of actual programs, and a parental population that resulted from selfing of a hybrid between inbred lines, which did not correspond to existing oil palm breeding populations. Moreover, Wong and Bernardo [14] only studied the selection response, without considering the evolution of genetic parameters. New studies on the potential of GS in oil palm would still be warranted, notably to cover more complex and general situations, and over several generations.

The aim of our study was to quantify the potential of RRGS as an alternative to conventional RRS, when the objective is to improve hybrid performance resulting from the multiplicative interaction between additive antagonistic components. We thus focused on the oil palm species, for which current conventional breeding is characterized by a long generation interval, due to progeny-tests, and by small populations. We simulated two realistic (complex) oil palm breeding populations with complementary characteristics for bunch production components (bunch weight [BW] and bunch number [BN]). We used these populations to compare several RRGS breeding schemes to traditional RRS over four generations, with the aim of improving the hybrid performance of interpopulation crosses for bunch production. More precisely, the simulations investigated the effects of four GS parameters on the selection response obtained with GBLUP in terms of bunch production: (1) the molecular data and associated GS model: in RRGS_PAR, we used parental genotypes to compute ***G*** matrices specific to each parental population and a model predicting two independent random effects of general combining abilities, one for each parental population, and in RRGS_HYB we used genotypes of both parents and hybrid individuals to compute one ***G*** matrix taking into account the parental origin of marker alleles and a model predicting a single random effect of breeding values; (2) the frequency of progeny tests (i.e. GS model calibration): every generation, every two generations or every four generations; (3) the number of candidate individuals: 120 or 300; and (4) for RRGS_HYB, the number of genotyped hybrids: 300, 1000 or 1700. We also studied the evolution of the genetic parameters in the parental populations: selection accuracy and additive variance for BW and BN, their genetic correlation and inbreeding.

## Methods

### Simulation overview

**Fig. 1:**
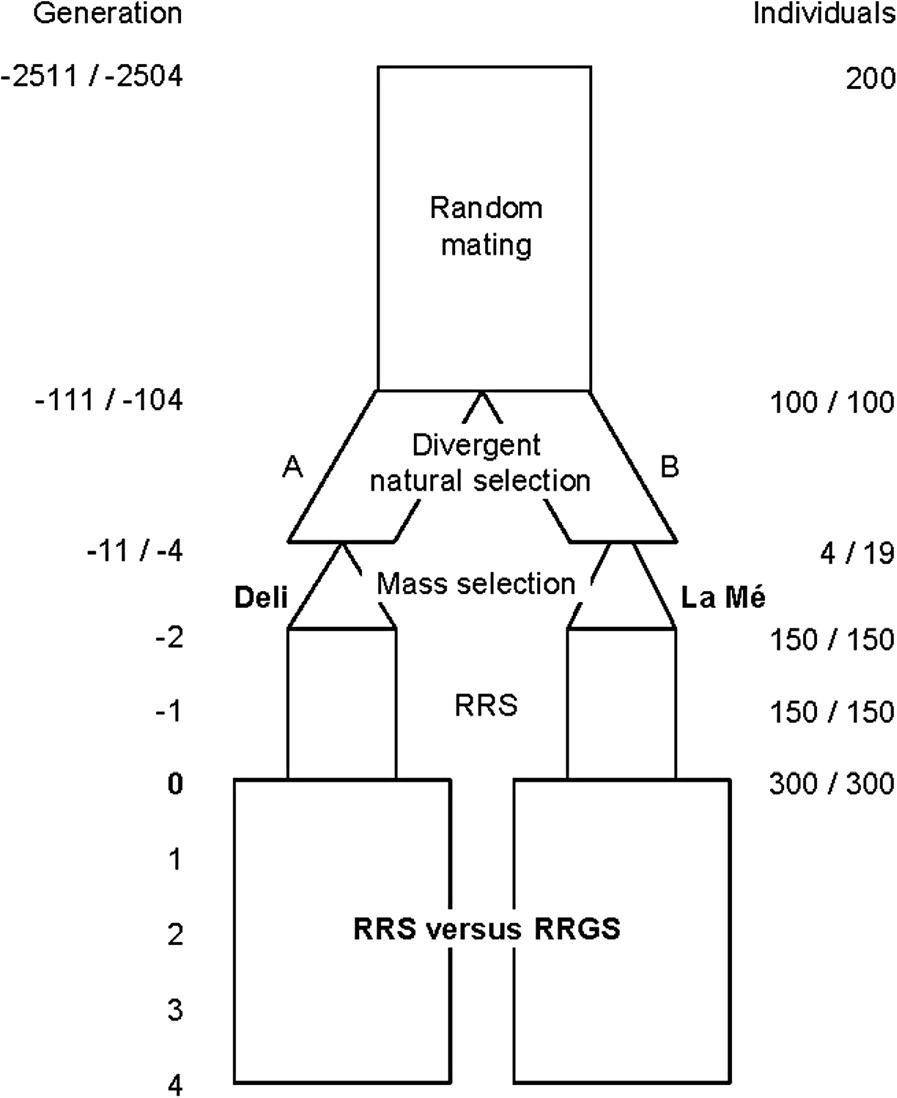
Simulation process to create two heterotic populations (similar to the actual Deli and La Mé oil palm breeding populations) and to compare reciprocal recurrent selection (RRS) and reciprocal recurrent genomic selection (RRGS) over four generations. Random mating allowed mutation-drift equilibrium to be achieved. Natural selection was applied to increase the bunch weight in population A and the bunch number in population B. Deli and La Mé populations originated from bottleneck events. In subsequent generations, artificial selection (mass selection, RRS or RRGS) was applied to increase bunch production, which is the bunch weight × bunch number product. Marker alleles were simulated from the start and QTL for bunch weight and bunch number were assigned after the first 2400 generations of random mating. RRS: reciprocal recurrent selection, RRGS: reciprocal recurrent genomic selection

The overall simulation process is summarized in Fig. 1. It involved three steps: (i) simulation of an equilibrium base population, (ii) simulation of initial breeding populations derived from this base population and having realistic genetic characteristics compared to current real oil palm breeding populations, and (iii) simulation of the breeding strategies (reciprocal recurrent selection [RRS] and two reciprocal recurrent genomic selection strategies [RRGS_PAR and RRGS_HYB]) applied to the initial breeding populations for four generations. The simulated genome had a length of 17 M and 16 chromosomes. The mutation rate was 10^−5^ per bp per meiosis and was kept constant throughout the simulation.

The simulations were performed with R software version 3.0.2 [15] and the HaploSim package [16]. The following paragraphs explain the three simulation steps in detail.

### Simulation of the equilibrium base population

We simulated a population over 2400 discrete generations with a constant size of 200 individuals having an equal contribution to the following generation and reproducing by random mating with the exclusion of selfing. In the first generation, 20000 bi-allelic loci (SNP) with equal distances between adjacent loci and equifrequent 0 and 1 alleles per locus were simulated across the genomes. The functions in HaploSim allowed simulation of meiosis between two parental haplotypes in order to produce a new individual. Mutation-drift equilibrium was assessed in the final generation with the distribution of allelic frequencies expected to follow a typical U-shaped curve. There were 17572 segregating SNP in the last generation. A single base population was generated this way and used as a starting point for the rest of the simulation process.

In the last generation, segregating SNP with a minor allele frequency (MAF) above 0.1 were chosen at random to be causative mutations (QTL). We assumed that the negative genetic correlation existing between BW and BN resulted from pleiotropy and consequently some QTL were randomly chosen to have pleiotropic effects. As the QTL number (*n*_*QTL*_) and percentage of pleiotropic QTL (*p*_*QTL*_) are unknown, we considered a range of values for these two parameters, assuming that this would include the true value of *n*_*QTL*_ and *p*_*QTL*_. We simulated *n*_*QTL*_ = 100, 500 and 1000 QTL per trait and *p*_*QTL*_ = 60, 75 % and 90 %. We assumed a simple additive architecture for BW and BN. For pleiotropic QTL, the QTL substitution effects for BW (*α*_*BW*_) and BN (*α*_*BN*_) were drawn from a normal bivariate distribution. This distribution was defined assuming a correlation of -0.9 and QTL variance equal to *σ*^2^_*a*(*BW*) *BP*_ / *n*_*QTL*_ for BN and to *σ*^2^_*a*(*BN*) *BP*_ / *n*_*QTL*_ for BW, with base population variances *σ*^2^_*a*(*BW*) *BP*_ = 6 and *σ*^2^_*a*(*BN*) *BP*_ = 12 chosen by trial and error so that *σ*^2^_*a*(*BW*)_ and *σ*^2^_*a*(*BN*)_ in the simulated initial breeding populations matched the actual values. For non-pleiotropic QTL, *α*_*BN*_ and *α*_*BW*_ were drawn from normal distributions using the same QTL variances as for pleiotropic QTL. The breeding (additive) value for each individual and the additive variance were defined according to the quantitative genetic model of Falconer and Mackay [4]. As we considered bi-allelic QTL, the breeding value for a trait at a QTL in a given population was equal to *−*2*pα* for homozygous genotype 00, (1 *−* 2*p*) *α* for heterozygote 01 and 2(1 *- p*) *α* for homozygote 11, where *p* was the frequency of allele 1 in the population and *α* the substitution effect of the QTL for the corresponding trait. The breeding value of each individual was obtained by summing across all QTL the breeding value at each QTL. In a given population, the intrapopulation additive variances were *σ*^2^_*a*(*BW*)_ = *∑*_*QTL*(*BW*)_ 2*p*(1 *– p*) *α*_*BW*_^2^ and *σ*^2^_*a*(*BN*)_ = *∑*_*QTL*(*BN*)_ 2*p*(1 *– p*) *α*_*BN*_^2^. As we assumed purely additive genetic determinism, they were equal to twice the interpopulation additive variances. The residual variances *σ*^2^_*e*(*BN*)_ and *σ*^2^_*e*(*BW*)_ were calculated with the formula *σ*^2^_*e*(*BW*)_ *= σ*^2^_*a*(*BW*)_ (1 *- h*^2^_*BP*_) */ h*^2^_*BP*_ and *σ*^2^_*e*(*BN*)_ *= σ*^2^_*a*(*BN*)_ (1 *- h*^2^_*BP*_) */ h*^2^_*BP*_ using base population heritability *h*^2^_*BP*_ = 0.8 for each trait, chosen so that *h*^2^ in the simulated initial breeding populations matched the actual *h*^2^. Environmental effects on BW and BN were generated from normal distributions with mean zero and variances *σ*^2^_*e*(*BW*)_ and *σ*^2^_*e*(*BN*)_. We assumed that *σ*^2^_*e*(*BW*)_ and *σ*^2^_*e*(*BN*)_ were the same for the two populations and they were kept constant throughout the simulation. The residual correlation between BW and BN was assumed to be zero. The phenotype was assumed to be the sum of the breeding value, environmental effects and mean value of the population. Initially, the mean values of the population for BW (kg) and BN traits were set at 15 in order to avoid negative values for bunch production and to obtain realistic phenotypic values for BW and BN in the simulated initial breeding populations.

### Simulation of initial breeding populations

The simulation process adopted to create the initial breeding populations from the equilibrium base population aimed to mimic what is known of the history of the Deli and La Mé populations [10, 17]. We proceeded by trial and error to set the simulation parameters that were not known from the literature or from real data (e.g. the selection intensity for mass selection), in order to generate breeding populations with genetic parameters that were consistent with actual parameters.

The equilibrium base population was randomly divided into two populations A and B of 100 individuals each. For 100 generations, A and B populations evolved independently and their sizes remained constant. Mating was random without selfing. Each population had a different selection regime so they had divergent evolution for the two traits: increasing BW in A and increasing BN in B. The parents of individuals in a given generation were sampled in the previous generation, where the probability for each individual to be chosen as parent was proportional to its phenotypic value.

After these first 100 generations, four individuals were taken at random in population A to simulate the bottleneck event at the origin of the actual Deli population, which originated from four oil palms planted in Indonesia in 1848. This was followed by three generations of random mating without selfing with 25 individuals per generation, and by six generations with an increasing number of individuals (50, 50, 60, 75, 100 and 150 individuals per generation). Mass selection was applied on bunch production during these last six generations. For mass selection, bunch production of each individual was computed as the product between BN and BW phenotypes. The best 70 % individuals were selected and randomly mated, with the exclusion of selfings, to produce the following generation. Similarly, in population B, after the first 100 generations of divergence, 19 individuals were taken at random to simulate the bottleneck event at the origin of the actual La Mé population in Côte d’Ivoire in the 1920s. This was followed by two generations with an increasing number of individuals (75, 150) and mass selection on bunch production. Mass selection was implemented in the same way as for Deli, but the top 30 % individuals were selected.

At that point, the simulated Deli and La Mé populations were submitted to two generations of RRS for bunch production, i.e. simulating what occurred in the real oil palm breeding populations from the 1950s. The principle of RRS is to select among candidate individuals based on their EBV, obtained from progeny tests. Here, EBV were simulated as values correlated with the true breeding values, with a correlation of 0.8 in the first RRS cycle and 0.9 in the second RRS cycle, corresponding to the accuracy of actual oil palm progeny tests. In each parental population, we selected the top 20 individuals giving crosses with highest expected bunch production. The expected bunch production of each cross between the progeny-tested Deli and La Mé was calculated as the product of the mean parental EBV for BW and BN. In each population, selected individuals reproduced by random mating with selfings allowed according to a diallel design in which 80 % of crosses were made. In the last generation (generation 0 in Fig. 1), 300 individuals were produced per population, uniformly distributed among crosses. They are hereafter referred to as initial breeding populations. Genotypes at SNP and QTL from the initial breeding populations, as well as pedigree information of the last four generations in Deli and last two in La Mé, were retained to be used in the final step of the simulation (comparison of RRS and RRGS).

The simulation process was repeated several times from the allocation of QTL to the generation of initial breeding populations. Runs were kept only if the genetic parameters in the simulated initial breeding populations were close to the real values of the current Deli and La Mé breeding populations. This calibration was done on: the fixation index *F*_*st*_ between Deli and La Mé, profiles of linkage disequilibrium (LD), narrow-sense heritabilities (*h*^2^), additive variances for BW and BN, and genetic correlations between BW and BN. Tables 1 and 2 summarize the observed values, mean values and standard deviations (SD) obtained for the different genetic parameters in the replicates kept for the study (five replicates per combination of *n*_*QTL*_ and *p*_*QTL*_). The real values of the *F*_*st*_, interpopulation additive variances and genetic correlations were obtained from the dataset described in Cros et al. [12]. It consisted of 131 Deli crossed with individuals from various African populations, including 94 La Mé, in order to progeny test them at Aek Loba (Sumatra). They were genotyped with 265 SSR markers. The Weir and Cockerham estimate of *F*_*st*_ was computed using the Hierfstat R package [18] in the simulated data, and with the diveRsity R package [19] in the real data. Although the simulated populations included SNP markers, the real value found with SSR markers could be used to calibrate the simulations, as the SSR had polymorphisms close to those of SNP. The actual genetic correlation between BN and BW and additive variances for BN and BW in the parental populations were computed from the hybrid phenotypic values using a mixed model analysis. For LD, the absolute values were affected by the marker type, so we only used the profile of LD curves to compare the simulated and real populations. As references, we used the LD curves calculated by Cochard [17], which showed higher LD in Deli over short distances (below 30–35 cM) and higher LD in La Mé for longer distances. For *h*^2^, as target values we used the mean *h*^2^ for BN and BW in Deli and La Mé reported in the literature [20–23]. Moreover, simulation runs where a single QTL explained over 20 % of the total additive variance were discarded, as this was considered unrealistically high. Finally, five replicates were produced for each combination of number of QTL (*n*_*QTL*_) and percentage of pleiotropic QTL (*p*_*QTL*_).

**Table 1:** Genetic parameters in the initial Deli and La Mé breeding populations (generation 0) obtained by simulation. Values are means over five replicates ± SD

		*Real*	*Number of QTL and percentage of pleiotropic QTL*		
		*values*		100	
			60 *%*	75 *%*	*9*0 *%*
*Fst*		0.49	0.49 ± 0.01	0.48 ± 0.03	0.47 ± 0.02
*LD* (*cM*)^a^					
*Deli*			4.9 ± 0.25	4.91 ± 0.07	5.05 ± 0.34
*La Mé*			2.44 ± 0.37	2.63 ± 0.27	2.73 ± 0.27
*h* ^2^					
*La Mé*	*BN*	0.56	0.63 ± 0.06	0.64 ± 0.04	0.64 ± 0.07
	*ABW*	0.56	0.65 ± 0.02	0.65 ± 0.04	0.63 ± 0.03
*Deli*	*BN*	0.56	0.57 ± 0.03	0.63 ± 0.07	0.6 ± 0.04
	*ABW*	0.56	0.54 ± 0.04	0.63 ± 0.06	0.58 ± 0.05
*True breeding values*					
*Deli*	*BN*		12.51 ± 0.5	11.05 ± 1.22	9.43 ± 0.87
	*ABW*		23.32 ± 0.9	22.18 ± 0.58	21.88 ± 0.5
*La Mé*	*BN*		25.27 ± 1.07	24.14 ± 0.97	24.01 ± 0.56
	*ABW*		12.71 ± 1.28	12.57 ± 0.87	10.85 ± 1.04
*Genetic correlation r*(*BN, ABW*)^b^					
*Deli*		−0.9	−0.69 ± 0.07	−0.75 ± 0.07	−0.9 ± 0.02
*La Mé*		−1.0	−0.73 ± 0.04	−0.73 ± 0.1	−0.86 ± 0.03
*Total intrapopulation additive variance*					
*Deli*	*BN*	5.5	1.55 ± 0.22	1.86 ± 0.63	1.78 ± 0.24
	*ABW*	2	0.76 ± 0.15	0.98 ± 0.38	0.87 ± 0.15
*La Mé*	*BN*	5	2 ± 0.37	1.82 ± 0.28	2.12 ± 0.5
	*ABW*	3	1.16 ± 0.2	1.04 ± 0.22	1.06 ± 0.2
*Mean intrapopulation additive variance at QTL* (*in % total*)					
*Deli*	*BN*		1.59 ± 0.1	1.57 ± 0.16	1.64 ± 0.16
	*ABW*		1.51 ± 0.03	1.59 ± 0.11	1.64 ± 0.17
*La Mé*	*BN*		1.41 ± 0.1	1.49 ± 0.06	1.45 ± 0.09
	*ABW*		1.4 ± 0.11	1.46 ± 0.07	1.48 ± 0.11
*Inbreeding*					
*Deli*			0.26 ± 0	0.26 ± 0	0.25 ± 0.01
*La Mé*			0.14 ± 0.01	0.13 ± 0.01	0.13 ± 0

^a^mean distance (cM) where linkage disequilibrium (LD) measured by *r* ^2^ between adjacent loci was 0.1, calibration was made on the shape of the curves

^b^the residual correlation in the real dataset was estimated at −0.15 and was considered to be 0 in the simulation

**Table 2:** Genetic parameters in the initial Deli and La Mé breeding populations (generation 0) obtained by simulation. Values are means over five replicates ± SD

		*Number of QTL and percentage of pleiotropic QTL*					
			500			1000	
		60 *%*	75 *%*	*9*0 *%*	60 *%*	75 *%*	*9*0 *%*
*Fst*		0.47 ± 0.03	0.47 ± 0.01	0.48 ± 0.02	0.49 ± 0.02	0.48 ± 0.01	0.47 ± 0.03
*LD* (*cM*)^a^							
*Deli*		4.47 ± 0.13	4.84 ± 0.34	4.59 ± 0.24	4.38 ± 0.12	4.18 ± 0.29	4.36 ± 0.3
*La Mé*		2.54 ± 0.15	2.83 ± 0.33	2.46 ± 0.31	2.31 ± 0.3	2.16 ± 0.24	2.27 ± 0.23
*h* ^2^							
*La Mé*	*BN*	0.67 ± 0.02	0.67 ± 0.01	0.66 ± 0.02	0.66 ± 0.01	0.66 ± 0.02	0.68 ± 0.01
	*ABW*	0.68 ± 0.01	0.67 ± 0.01	0.67 ± 0.01	0.67 ± 0.01	0.67 ± 0.01	0.69 ± 0.01
*Deli*	*BN*	0.63 ± 0.01	0.63 ± 0.01	0.64 ± 0.03	0.64 ± 0.02	0.65 ± 0.02	0.64 ± 0.02
	*ABW*	0.61 ± 0.03	0.63 ± 0.02	0.64 ± 0.04	0.63 ± 0.02	0.65 ± 0.02	0.64 ± 0.01
*True breeding values*							
*Deli*	*BN*	12.47 ± 0.84	11.73 ± 1.62	10.11 ± 0.74	12.41 ± 1.51	10.52 ± 0.57	10.14 ± 0.98
	*ABW*	23 ± 0.3	21.09 ± 1.11	21.53 ± 0.57	23.11 ± 1.05	22.45 ± 0.41	21.63 ± 0.39
*La Mé*	*BN*	25.69 ± 0.93	24.76 ± 0.4	24.42 ± 0.75	26.43 ± 1.47	25.92 ± 0.57	24.81 ± 1.35
	*ABW*	13.54 ± 0.56	12.65 ± 0.6	11.44 ± 0.45	12.65 ± 1.37	12.19 ± 0.93	11.26 ± 0.33
*Genetic correlation r*(*BN, ABW*)^a^							
*Deli*		−0.64 ± 0.05	−0.79 ± 0.05	−0.83 ± 0.03	−0.64 ± 0.08	−0.75 ± 0.07	−0.84 ± 0.04
*La Mé*		−0.68 ± 0.03	−0.73 ± 0.03	−0.86 ± 0.04	−0.66 ± 0.01	−0.71 ± 0.05	−0.81 ± 0.03
*Total interpopulation additive variance*							
*Deli*	*BN*	1.94 ± 0.13	1.99 ± 0.14	2.03 ± 0.42	1.94 ± 0.1	1.99 ± 0.13	1.97 ± 0.18
	*ABW*	0.91 ± 0.16	0.94 ± 0.08	1 ± 0.21	0.96 ± 0.05	0.99 ± 0.06	0.93 ± 0.05
*La Mé*	*BN*	2.27 ± 0.14	2.27 ± 0.09	2.27 ± 0.3	2.2 ± 0.06	2.11 ± 0.19	2.36 ± 0.15
	*ABW*	1.21 ± 0.03	1.11 ± 0.07	1.15 ± 0.13	1.14 ± 0.08	1.08 ± 0.1	1.16 ± 0.06
*Mean interpopulation additive variance at QTL* (*in % total*)							
*Deli*	*BN*	0.32 ± 0.01	0.31 ± 0.01	0.31 ± 0.02	0.16 ± 0	0.15 ± 0.01	0.16 ± 0
	*ABW*	0.32 ± 0.01	0.31 ± 0.01	0.31 ± 0.02	0.16 ± 0	0.16 ± 0	0.16 ± 0
*La Mé*	*BN*	0.29 ± 0.01	0.29 ± 0.01	0.28 ± 0.01	0.14 ± 0	0.14 ± 0	0.14 ± 0
	*ABW*	0.29 ± 0.01	0.29 ± 0.01	0.28 ± 0.01	0.15 ± 0	0.14 ± 0.01	0.14 ± 0
*Inbreeding*							
*Deli*		0.26 ± 0.01	0.26 ± 0.01	0.25 ± 0	0.26 ± 0	0.25 ± 0.01	0.26 ± 0.01
*La Mé*		0.13 ± 0.01	0.14 ± 0.01	0.13 ± 0	0.14 ± 0	0.14 ± 0.01	0.13 ± 0.01

^a^the residual correlation in the real dataset was estimated at −0.15 and was considered to be 0 in the simulation

### Simulation of reciprocal recurrent selection and reciprocal recurrent genomic selection

The initial Deli and La Mé breeding populations were used as starting points to compare conventional RRS with RRGS over four generations, in terms of the genetic gain for bunch production in hybrid individuals (selection response) and the evolution of genetic parameters in the parental populations (selection accuracy and additive variance for BN and BW, genetic correlation between BN and BW, inbreeding).

In reciprocal recurrent selection (RRS), the EBV of the Deli and La Mé selection candidates were obtained from the analysis of their progeny tests. At each generation, the progeny tests involved 120 Deli and 120 La Mé. The mating design for progeny tests was an incomplete factorial design with 300 crosses (i.e. 2.5 crosses per parent, with variations in the number of crosses per parent kept as small as possible). The chosen numbers of progeny-tested individuals and crosses corresponded to what is done in actual oil palm breeding programs. We simulated 45 individuals per cross, along with their resulting SNP genotypes, QTL genotypes and breeding and phenotypic values for BW and BN. The progeny tests were analyzed with a bivariate mixed model to obtain the EBV of the 120 Deli and 120 La Mé for BW and BN. The model was of the following form:

$$ \left[\begin{array}{c}\hfill {y}_{BW}\hfill \\ {}\hfill {y}_{BN}\hfill \end{array}\right]=\left[\begin{array}{c}\hfill {\mu}_{BW}\hfill \\ {}\hfill {\mu}_{BN}\hfill \end{array}\right]+\left[\begin{array}{c}\hfill {\boldsymbol{Z}}_{\boldsymbol{Deli}\left(\boldsymbol{B}\boldsymbol{W}\right)}\hfill \\ {}\hfill 0\hfill \end{array}\begin{array}{c}\hfill 0\hfill \\ {}\hfill {\boldsymbol{Z}}_{\boldsymbol{Deli}\left(\boldsymbol{B}\boldsymbol{N}\right)}\hfill \end{array}\right]\left[\begin{array}{c}\hfill {a}_{Deli(BW)}\hfill \\ {}\hfill {a}_{Deli(BN)}\hfill \end{array}\right]+\left[\begin{array}{c}\hfill {\boldsymbol{Z}}_{\boldsymbol{La}\ \boldsymbol{M}\acute{\boldsymbol{e}} \left(\boldsymbol{B}\boldsymbol{W}\right)}\hfill \\ {}\hfill 0\hfill \end{array}\begin{array}{c}\hfill 0\hfill \\ {}\hfill {\boldsymbol{Z}}_{\boldsymbol{La}\ \boldsymbol{M}\acute{\boldsymbol{e}} \left(\boldsymbol{B}\boldsymbol{N}\right)}\hfill \end{array}\right]\left[\begin{array}{c}\hfill {a}_{La\ M\acute{e}(BW)}\hfill \\ {}\hfill {a}_{La\ M\acute{e}(BN)}\hfill \end{array}\right]+\left[\begin{array}{c}\hfill {e}_{BW}\hfill \\ {}\hfill {e}_{BN}\hfill \end{array}\right] $$

with *y*_*BW*_ and *y*_*BN*_being vectors of phenotypic values of the 13,500 hybrid individuals for BW and BN, *μ*_*BW*_ and *μ*_*BN*_ being overall means of hybrid individuals for BW and BN, and *e*_*BW*_ and *e*_*BN*_ being vectors of residual effects for BW [*~ N*(0*,****I****σ*^2^_*e*(*BW*)_)] and BN [*~ N*(0*,****I****σ*^2^_*e*(*BN*)_)]. The vectors of EBV (actually general combining ability) of BW and BN in Deli *a*_*Deli*(*BW*)_ and *a*_*Deli*(*BN*)_ followed a bivariate normal distribution $$ N\left(0,\left(\begin{array}{cc}\hfill \sigma {{}^2}_{Del{i}_{(BW)}}\hfill & \hfill {\sigma}_{Del{i}_{\left(BN,BW\right)}}\hfill \\ {}\hfill {\sigma}_{Del{i}_{\left(BN,BW\right)}}\hfill & \hfill \sigma {{}^2}_{Del{i}_{(BN)}}\hfill \end{array}\right)\otimes 0.5{\boldsymbol{A}}_{\boldsymbol{Deli}}\right), $$ with *σ*_*Deli*(*BN,BW*)_being the additive covariance between BN and BW. The vectors of general combining ability of La Mé *a*_*La Mé*(*BW*)_ and *a*_*La Mé*(*BN*)_ had a distribution similar to that of the population specific parameters ***A***_***La Mé***_, *σ*^2^_*La Mé*(*BW*)_, *σ*^2^_*La Mé*(*BN*)_ and *σ*_*La Mé*(*BN, BW*)_. ***A***_***Deli***_ and ***A***_***La Mé***_ were matrices of additive relationships among Deli and La Mé individuals computed from pedigrees. ***Z***_***Deli*(*BW*)**_, ***Z***_***Deli*(*BN*)**_, ***Z***_***La Mé*(*BW*)**_ and ***Z***_***La Mé*(*BN*)**_ were incidence matrices and ***I*** was an identity matrix. The R-ASReml package [24] was used to obtain variance component estimates and EBV of Deli and La Mé individuals. In each population, the best 20 individuals giving the crosses with highest expected bunch production were selected based on their EBV, as described for the previous step of the simulation, and they reproduced by random mating with selfings allowed according to a half diallel design in which 80 % of crosses were made (consequently, 168 different within-population crosses could be made). The number of crosses was the same for all individuals. 120 progenies per population were produced. The generation interval for RRS was 20 years.

Reciprocal recurrent genomic selection (RRGS) gave genomic estimated breeding values (GEBV) of the Deli and La Mé selection candidates, from an analysis combining their genotype and their progeny tests or from their sole genotype. As in RRS, the progeny tests involved 120 Deli and 120 La Mé. GEBV were obtained using 2500 SNP markers with MAF > 4 % and the GBLUP statistical method. In the simulations, we studied the effects of reducing the generation interval and increasing the selection intensity on the RRGS performance. First, a reduction in the generation interval was obtained when applying RRGS in the generation(s) following the progeny-tested individuals. In this case, the selection candidates were not progeny tested but only genotyped; and they were selected based on their sole genotype and reproduced once they were sexually mature. We considered the generation interval was consequently reduced to six years (instead of 20 years in the generations where progeny tests were performed, as in RRS). We varied the progeny-test frequency to assess the potential of RRGS when used to reduce the generation interval. They were simulated every generation, leading to a total number of 80 years to complete the four cycles, every two generations (52 years to complete four cycles) or every four generations (38 years to complete four cycles). The GEBV of the 120 Deli and 120 La Mé individuals in the generations with progeny tests were predicted from the phenotypic data of hybrid individuals and molecular data of either only Deli and La Mé individuals (RRGS_PAR, see model below) or Deli and La Mé individuals plus hybrid individuals (RRGS_HYB, see model below). The GEBV of the Deli and La Mé individuals in the generations without progeny tests were predicted in the same way, except that the phenotypic data used to calibrate the GS model were those from the last generation of progeny tests. Secondly, to study the effect of increasing the selection intensity, we also applied RRGS to a number of selection candidates (300 per population) larger than the number of progeny-tested individuals (120 per population). As 168 different within-population crosses could be made, the 300 individuals were obtained by simulating one or two individuals per possible cross, up to a total of 300. In the generations with progeny tests and when using 300 candidates, 120 individuals were randomly chosen to be progeny tested among the 300 and the selection was made among them and their 180 non-progeny-tested sibs.

In RRGS_PAR, the progeny tests were analyzed with the same bivariate model as for RRS, except that matrices of additive relationships ***A***_***Deli***_ and ***A***_***La Mé***_ were replaced by molecular relationship matrices ***G***_***Deli***_ and ***G***_***La Mé***_ computed from parental genotypes, using observed allele frequencies [2, 3] and normalized to have average diagonal coefficients equal to one [25]. Therefore, RRGS_PAR used two population-specific molecular relationship matrices.

In RRGS_HYB, the molecular relationship matrix ***G*** of the genotyped individuals (all the Deli and La Mé and the *n*_*genotyped*_ hybrid individuals) was computed using Deli and La Mé genotypes and hybrid gametotypes, taking the parental origin of marker alleles for hybrid individuals into account. Each SNP was thus converted into a multiallelic marker with alleles 0_Deli_, 1_Deli_, 0_La Mé_ and 1_La Mé_. From these molecular data, ***G*** was computed according to Van Raden [2] and Habier et al. [3], using observed allele frequencies and a modification implemented by Legarra (pers. comm.) for the multiallelic case, and it was normalized to have average diagonal coefficients equal to one [25]. The number of genotyped hybrid individuals was *n*_*genotyped*_ = 300, 1000 and 1700. The corresponding breeding strategies were named RRGS_HYB300, RRGS_HYB1000 and RRGS_HYB1700. The genotyped hybrid individuals were randomly sampled among the 13,500 existing hybrids, taking the same number of individuals per cross (i.e. one when *n*_*genotyped*_ = 300). As the progeny tests included 13,500 phenotyped hybrids, the non-genotyped hybrids were also included in the model, as their phenotypic values contributed to estimate the GEBV of their parents. For this purpose, we used the single-step approach of Legarra et al. [26], which involved combining the ***G*** matrix with the genealogical additive relationship matrix ***A***_***all***_ of all the Deli and La Mé individuals and all the hybrids, according to: $$ {\boldsymbol{H}}^{-1}={{\boldsymbol{A}}_{\boldsymbol{all}}}^{-1}+\left(\begin{array}{cc}\hfill 0\hfill & \hfill 0\hfill \\ {}\hfill 0\hfill & \hfill {\boldsymbol{G}}^{-1} - {{\boldsymbol{A}}_{22}}^{-1}\hfill \end{array}\right), $$ with ***A***_**22**_ being the matrix of the genealogical additive relationship matrix of the genotyped individuals (parents and hybrids). The bivariate model for RRGS_HYB was:

$$ \left[\begin{array}{c}\hfill {y}_{BW}\hfill \\ {}\hfill {y}_{BN}\hfill \end{array}\right]=\left[\begin{array}{c}\hfill {\mu}_{BW}\hfill \\ {}\hfill {\mu}_{BN}\hfill \end{array}\right]+\left[\begin{array}{c}\hfill {\boldsymbol{Z}}_{\boldsymbol{Deli}\left(\boldsymbol{B}\boldsymbol{W}\right)}\hfill \\ {}\hfill 0\hfill \end{array}\begin{array}{c}\hfill 0\hfill \\ {}\hfill {\boldsymbol{Z}}_{\boldsymbol{Deli}\left(\boldsymbol{B}\boldsymbol{N}\right)}\hfill \end{array}\right]\left[\begin{array}{c}\hfill {a}_{BW}\hfill \\ {}\hfill {a}_{BN}\hfill \end{array}\right]+\left[\begin{array}{c}\hfill {e}_{BW}\hfill \\ {}\hfill {e}_{BN}\hfill \end{array}\right] $$

The vectors *a*_*BN*_ and *a*_*BW*_ of breeding values of all individuals (hybrids and their parents) for BN and for BW followed $$ N\left(0,\left(\begin{array}{cc}\hfill \sigma {2}_{a_{(BW)}}\hfill & \hfill {\sigma}_{a_{\left(BN,BW\right)}}\hfill \\ {}\hfill {\sigma}_{a_{\left(BN,BW\right)}}\hfill & \hfill \sigma {2}_{a_{(BN)}}\hfill \end{array}\right)\otimes \boldsymbol{H}\right), $$ with *σ*^2^_*a*(*BN*)_ and *σ*^2^_*a*(*BW*)_ being the additive variances and *σ*_*a*(*BN, BW*)_ being the additive covariance between BN and BW.

We did not consider genotyping more than 1700 hybrid individuals or using more than 300 candidate individuals per population for computational reasons.

### Analysis of results

We distinguished between two types of factors: technical factors under the breeder’s control (breeding strategy [RRS, RRGS_PAR and RRGS_HYB], number of selection candidates, frequency of progeny tests and number of genotyped hybrids); and the biological factors defining the genetic architecture of the traits under selection (number of QTL, percentage of pleiotropic QTL). The effects of biological factors and the interaction between biological and technical factors are crucial, because the genetic architecture is unknown in actual situations. The breeder has to design a breeding program where technical factors will give the highest annual selection response, regardless of the unknown genetic architecture of the traits.

We defined a breeding scheme as a combination of breeding strategy (RRS, RRGS_HYB and RRGS_PAR), frequency of progeny tests (every generation, every two or every four generations), number of selection candidates (120 or 300) and number of genotyped hybrid individuals (0, 300, 1000 and 1700). Each of these combinations had five replicates, which had different simulated initial breeding populations.

At the end of the simulation (i.e. in generation 4 of Fig. 1), we measured the cumulative selection response in hybrid individuals, expressed as a percentage of hybrid production in the initial generation (generation 0), and the annual selection response in hybrid individuals, which was the cumulative response divided by the number of years required to carry out the four breeding generations. Analyses of variance (ANOVA) were performed to study the effect of the different technical and biological factors and their interactions on the selection response, as well as on the genetic parameters in the parental populations (selection accuracy and additive variance for BW and BN, genetic correlation between BW and BN, inbreeding). The means for the levels of factors in the ANOVA were compared using the Tukey’s honest significant difference method.

## Results

### Number of genotyped hybrids in RRGS_HYB

In order to simplify the interpretation of the results, we first focused on the number of genotyped hybrid individuals, as it had a major effect on the annual selection response in RRGS_HYB. Table 3 presents the ranking of the breeding schemes according to their annual selection response, and shows that the annual response of RRGS_HYB increased with the number of genotyped hybrids. RRGS_HYB could outperform RRS when 1000 or 1700 hybrids were genotyped. The difference between RRGS_HYB1700 and RGGS_HYB1000 was generally small but was always to the advantage of RRGS1700 across all combinations. RRGS_HYB with 300 genotyped hybrids was worse than the other two alternatives, with 1000 and 1700, as its annual response was always lower or equal to RRGS_PAR and RRS. Therefore we only considered the RRGS_HYB1700 results for the rest of the study.

**Table 3:** Ranking of breeding schemes according to their mean annual response

Rank	Breeding strategy	Progeny test frequency	Number of candidates	Number of genotyped hybrids	Annual response (%)	Change compared to RRS (%)
1	RRGS**_HYB**	GMMM	300	1700	0.45 a	71.8 %
2	RRGS**_HYB**	GMMM	300	1000	0.41 b	53.8 %
3	RRGS**_HYB**	GMMM	120	1700	0.39 bc	47.7 %
4	RRGS_PAR	GMMM	300		0.38 bcd	45.7 %
5	RRGS_PAR	GMGM	300		0.38 bcd	45.0 %
6	RRGS**_HYB**	GMGM	300	1700	0.36 cde	38.3 %
7	RRGS**_HYB**	GMGM	300	1000	0.36 cde	38.2 %
8	RRGS**_HYB**	GMMM	120	1000	0.35 de	34.2 %
9	RRGS_PAR	GMMM	120		0.34 e	30.7 %
10	RRGS_PAR	GMGM	120		0.34 ef	27.6 %
11	RRGS**_HYB**	GMGM	120	1700	0.33 ef	25.8 %
12	RRGS**_HYB**	GMGM	120	1000	0.31 fg	17.3 %
13	RRGS_PAR	GGGG	300		0.28 gh	7.7 %
14	RRGS**_HYB**	GMGM	300	300	0.28 gh	6.3 %
15	RRGS**_HYB**	GGGG	300	1700	0.28 gh	5.1 %
16	RRGS**_HYB**	GMMM	300	300	0.27 hi	3.4 %
17	RRGS_PAR	GGGG	120		0.26 hi	0.1 %
*18*	*RRS*	*GGGG*	*120*		*0.26 hi*	*0.0 %*
19	RRGS**_HYB**	GGGG	300	1000	0.26 hi	−0.5 %
20	RRGS**_HYB**	GMMM	120	300	0.25 hij	−4.3 %
21	RRGS**_HYB**	GGGG	120	1700	0.24 ijk	−8.3 %
22	RRGS**_HYB**	GMGM	120	300	0.24 ijk	−9.0 %
23	RRGS**_HYB**	GGGG	120	1000	0.22 jk	−15.2 %
24	RRGS**_HYB**	GGGG	300	300	0.21 kl	−20.6 %
25	RRGS**_HYB**	GGGG	120	300	0.18 l	−32.8 %

The annual response is expressed in percentage of hybrid production in the initial generation (generation 0) per year. Breeding scheme includes the breeding strategy (*RRS*: reciprocal recurrent selection, RRGS: reciprocal recurrent genomic selection), individuals genotyped to calibrate the GS model (_PAR: genotyping only parents of progeny tests when calibrating the GS model, **_HYB**: genotyping, in addition, hybrid individuals), number of candidates per population and generation (120 and 300, in RRS the set of candidates is limited to the 120 progeny-tested individuals of each parental population), progeny test frequency (GGGG: every generation, GMGM: every two generations and GMMM: every four generations) and for RRGS_HYB number of genotyped hybrids (300, 1000 and 1700). Values are means over 45 replicates (3 numbers of QTL × 3 percentage of pleiotropic QTL × 5 replicates). Values with the same letter are not significantly different at *P* = 0.001

### Selection accuracy

The selection accuracy with RRS was very high and remained constant over generations, i.e. around 0.967 ± 0.003 (SD), with a negligible effect of trait and population (not shown).

**Fig. 2:**
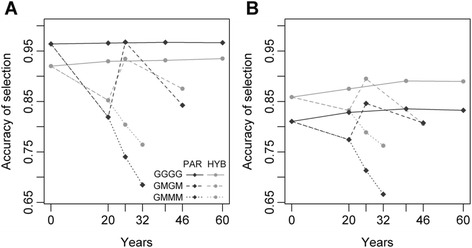
Accuracy of reciprocal recurrent genomic selection (RRGS) for bunch number in the Deli population according to years and the RRGS breeding scheme with (**a**) 120 and (**b**) 300 selection candidates. The breeding scheme includes individuals genotyped to calibrate the GS model (parents and 1700 hybrids in RRGS_HYB and only parents in RRGS_PAR) and the progeny test frequency (GGGG: every generation, GMGM: every two generations and GMMM: every four generations). Means are calculated over 45 values

For RRGS, we first considered its simplest implementation i.e. when calibrating the GS model every generation and using sets of candidate individuals limited to the 120 progeny-tested individuals. In this case, the accuracy of RRGS_PAR (0.968 ± 0.008) was similar to that of RRS, while that of RRGS_HYB1700 was slightly but significantly less good (*P* < 0.001), with an accuracy of 0.934 ± 0.008 (see Fig. 2 concerning the example of BN in Deli). Secondly, we assessed how the selection accuracy was affected by the absence of progeny tests. The accuracy of the progeny-tested individuals was much higher than that of non-progeny-tested individuals of the following(s) generation(s), which fell to 0.748 ± 0.058 for RRGS_HYB and even lower for RRGS_PAR (0.615 ± 0.101). When three generations of selection were made without calibration of the GS model, the selection accuracy kept decreasing and this occurred at a higher pace for RRGS_PAR than for RRGS_HYB (Fig. 2). The accuracy of the progeny-tested individuals was also higher than that of their 180 non-progeny-tested sibs, which was 0.852 ± 0.019 for RRGS_HYB and 0.744 ± 0.020 for RRGS_PAR. This can be seen when comparing levels of equivalent combinations between Fig. 2a and b: the inclusion of 180 non-progeny tested individuals in the latter decreased the overall level of accuracy. For all non-progeny tested individuals, the accuracy was significantly lower with RRGS_PAR than with RRGS_HYB (*P* < 0.001).

The key results here were that the selection accuracy was reduced in individuals that were not progeny tested, and that the two RRGS methods behaved differently in terms of selection accuracy for progeny-tested individuals (RRGS_PAR slightly more accurate than RRGS_HYB) and non-progeny-tested individuals (RRGS_HYB much more accurate than RRGS_PAR).

### Additive variance

**Fig. 3:**
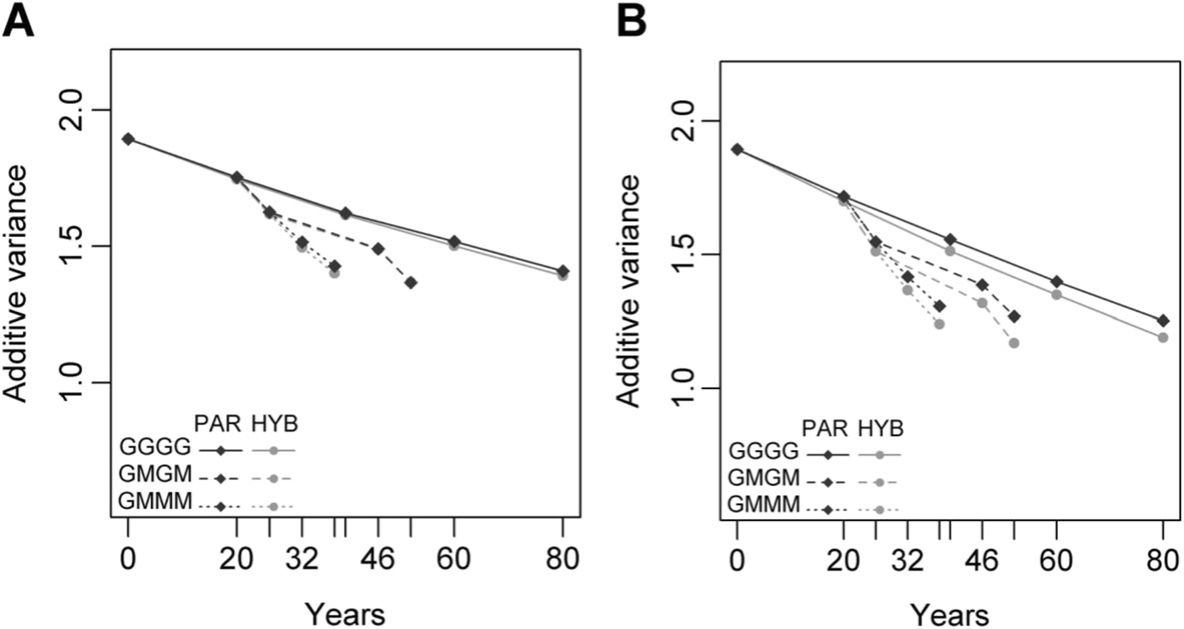
Additive variance for bunch number according to years and the reciprocal recurrent genomic selection (RRGS) breeding scheme in Deli with (**a**) 120 and (**b**) 300 selection candidates. The breeding scheme includes individuals genotyped to calibrate the GS model (parents and 1700 hybrids in RRGS_HYB, and only parents in RRGS_PAR) and the progeny test frequency (GGGG: every generation, GMGM: every two generations and GMMM: every four generations). Means are calculated over 45 values

The additive variance decreased with generations, which is a well-known effect of selection and genetic drift. The decrease in additive variance with RRS was identical to RRGS_PAR when using 120 candidates and calibrated every generation (not shown). Fig. 3 shows the results obtained with RRGS in Deli when using 120 and 300 candidates, with the example of BN (similar trends were obtained for both traits and both populations). The major factor affecting the cumulative decrease in additive variance was the number of candidates, with 300 resulting in a more rapid decrease in variance than with 120 candidates (see Fig. 3a versus Fig. 3b, where the additive variance decreased by 27 % after four generations with 120 candidates but by 35 % with 300 candidates, *P* < 0.001). A similar result was obtained for both populations and traits. On average, using 120 candidates decreased the additive variance in RRGS strategies by 32 % while using 300 individuals led to a decrease of 39.6 %. This occurred as the number of selected individuals was kept constant, so increasing the number of candidates led to the selection of individuals with a higher average value but lower genetic variability. We also noted that with 300 candidates the decrease in additive variance with RRGS_HYB after four generations (−41.3 %) was similar than with RRGS_PAR (−37.8 %), the difference being not significant. This indicated that the number of candidates was the major factor affecting the decrease in variation.

The number of QTL that were assumed in the model also had a role regarding the decrease in additive variance. In the same example (BN in Deli), the additive variance decreased by around 27 % with either 1000 or 500 QTL but by 37 % with 100 QTL (*P* < 0.001). This occurred as the simulation was designed to have the same additive variances in generation 0 regardless of the number of QTL. Consequently, when the QTL were fewer they also had stronger effects and were correspondingly under higher selection pressure up to their fixation. Therefore, the fewer the number of QTL, the stronger the effect of selection was in depleting the additive variance.

### Selection response

All the biological factors (number of QTL and percentage of pleiotropic QTL) had significant effects on the selection response at *P* < 0.001. The percentage of pleiotropic QTL was the most important factor of the study. The number of QTL also had a strong effect. The selection response increased when the percentage of pleiotropic QTL decreased and when the number of QTL increased. The cumulative response was 14.7, 18.6 % and 22 % with 90, 75 and 60 % pleiotropic QTL, respectively (*P* < 0.001 for all differences), and the annual response was 0.26, 0.33 and 0.40 % (*P* < 0.001). This resulted from the fact that the genetic progress potential depended mostly on the fixation of favorable alleles at QTL controlling either BN or BW, rather than at the pleiotropic QTL, as they had antagonistic effects on both traits. When the percentage of pleiotropic QTL decreased, the number of QTL controlling only BN or BW increased, therefore giving a higher genetic progress potential. Similarly, the genetic progress potential was higher when the total number of QTL controlling each trait increased.

The major technical factors affecting the selection response was the progeny-test frequency, followed by the number of candidates, the breeding strategy and, to a lesser extent, the interaction between the breeding strategy and the progeny-test frequency (*P* < 0.001). Their effects are detailed in the following paragraphs.

We first compared the selection response of RRS with RRGS strategies that differed with RRS only by the use of relationship matrices computed with markers instead of the pedigrees, i.e. considering only RRGS_HYB1700 and RRGS_PAR with calibration every generation and sets of candidates limited to 120 progeny-tested individuals. In this case, the selection response was similar for RRGS_HYB1700, RRGS_PAR and RRS (20.5 % over four generations, or 0.26 % per year, see Table 3). Therefore, obtaining a higher selection response with RRGS compared to RRS could not be achieved without modifying the breeding scheme in order to reduce the generation interval or to increase the selection intensity.

Secondly, to study the effect of reducing the generation interval in RRGS (by a decrease in the progeny-test frequency), we considered RRGS_HYB1700 and RRGS_PAR with calibration of the GS model every two or four generations and with the same number of candidates as in RRS (120). In this case, decreasing the progeny-test frequency led to a lower cumulative selection response, i.e. 13.9, 17.4 and 20.2 % with progeny tests performed every four generations, every two generations and every generation, respectively (all differences significant at *P* < 0.001). With generations without progeny tests, the selection accuracy was reduced and consequently the cumulative selection response decreased. An opposite effect was noted in the annual response, i.e. 0.37, 0.33 and 0.25 % with progeny tests performed every four generations, every two generations and every generation, respectively (*P* < 0.001). This was due to the favorable effect of the decreased frequency of progeny tests on the ratio between the selection accuracy and the generation interval (*r*_*AÂ*_*/L*), where the decrease in generation interval more than balanced out the decrease in accuracy, while the other factors (selection intensity, additive variance) remained unaltered. Indeed, performing progeny tests every two generations decreased the generation interval by 35 % compared to those performed every generation and decreased the selection accuracy to 0.90, which was 4 % lower than with progeny tests performed every generation and with 120 candidates for RRGS_HYB and 10 % lower for RRGS_PAR. With progeny tests performed every four generations, the generation interval decreased by 52.5 % and the selection accuracy dropped to 0.84 for RRGS_HYB (10 % decrease compared to progeny tests performed each generation and 120 candidates) and to 0.80 for RRGS_PAR (17 % decrease). Therefore when the GS model calibration frequency declined, the cumulative selection response was lower but the *r*_*AÂ*_*/L* ratio and annual selection response were higher. With the decrease in the progeny test frequency, the relative potential of RRGS_HYB and RRGS_PAR varied due to their different accuracy in generations with and without progeny tests. With calibration every generation, as the RRGS_HYB accuracy was lower than that of RRGS_PAR, the annual selection response of RRGS_HYB was lower than that of RRGS_PAR, although not significantly (Table 3). With progeny tests performed every two generations, as the RRGS_PAR accuracy decreased more than that of RRGS_HYB, both methods had the same annual selection response, which was significantly higher than the annual selection response of RRS. With progeny tests performed every four generations, RRGS_HYB finally outperformed RRGS_PAR in accuracy and had a significantly higher annual selection response than the other breeding schemes (+50 % compared to RRS for RRGS_HYB, +30 % for RRGS_PAR).

Thirdly, regarding the RRGS gain, we studied the effect of an increase in selection intensity, which was obtained by increasing the number of candidates, as the number of selected individuals was constant. With 120 candidates, the best 16.7 % individuals were selected, while with 300 candidates only the top 6.7 % were selected, which is a 2.5-fold more stringent selection intensity. This significantly increased the selection response, as a consequence of the effect of the number of candidates on the *r*_*ÂA*_ *× i × σ*_*a*_ product with the increase in *i* (×2.5) being much higher than the joint decrease in *r*_*ÂA*_ (−8.8 % in RRGS_HYB, *−*23.1 % in RRGS_PAR) and *σ*_*a*_ (−3.3 %). Again, due to the superiority of RRGS_HYB over RRGS_PAR in maintaining the selection accuracy for non-progeny-tested individuals, the increase in the number of candidates benefited RRGS_HYB more than RRGS_PAR (Table 3). The annual response was always higher with 300 candidates than with 120 candidates, but this difference was only significant for RRGS_HYB (+12.8 %, *P* < 0.001 for RRGS_HYB, +7.1 % for RRGS_PAR). We also found that the number of candidates significantly interacted with the percentage of pleiotropic QTL and the number of QTL on the selection response, although these interactions had less impact than the previously mentioned factors. This was not surprising as, with the largest numbers of non-pleiotropic QTL per trait (either due to a high number of QTL or to a low percentage of pleiotropic QTL), 120 selection candidates were not enough to capture all of the existing additive variation. In this case, using 300 candidates led to a higher selection response. By contrast, with a smaller number of non-pleiotropic QTL, 120 candidates were enough to capture all of the additive variation and an increase in the number of candidates did not markedly increase the selection response.

Finally, when RRGS was used to both decrease the generation interval and increase the selection intensity compared to RRS, the best breeding scheme was RRGS_HYB1700 with progeny tests conducted every four generations and 300 candidates, with an annual selection response of 0.45 % per year, i.e. 71.8 % higher than RRS and significantly higher than all other breeding schemes at *P* < 0.001 (Table 3).

**Fig. 4:**
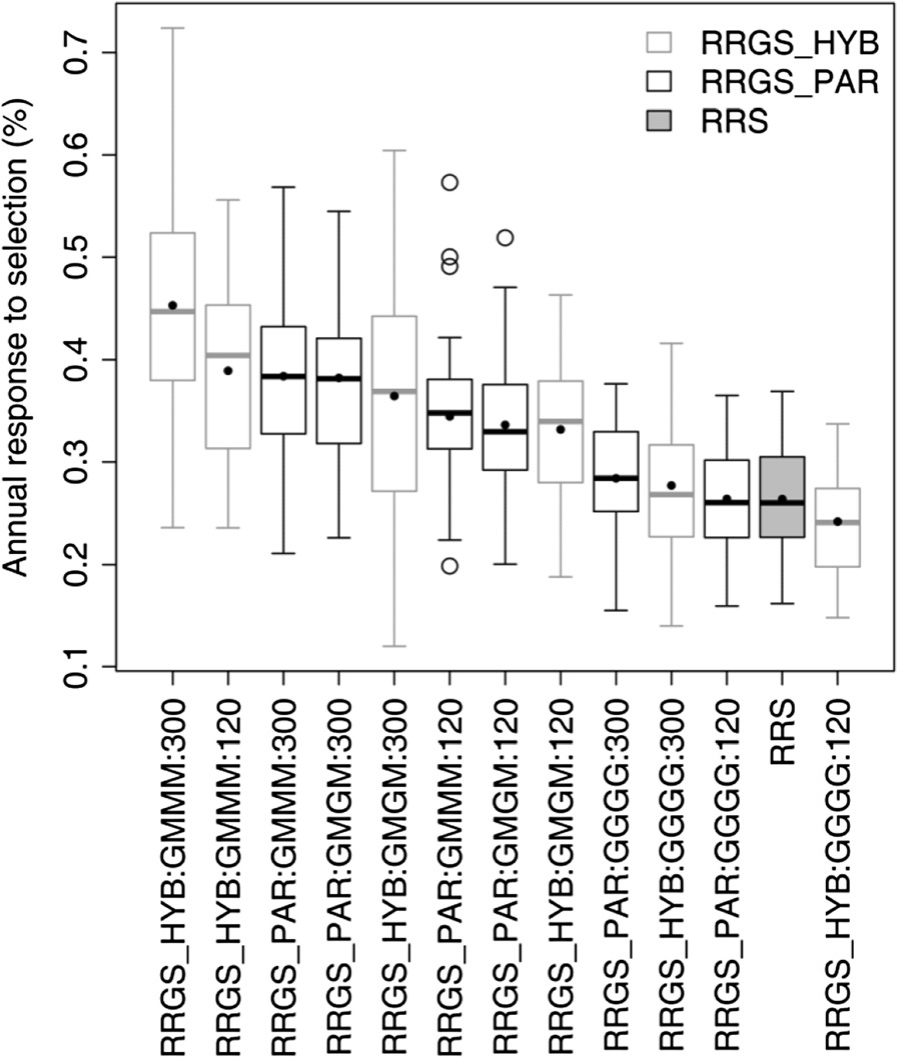
Variation in annual selection response associated with each breeding scheme. The breeding scheme includes the breeding strategy (RRS: reciprocal recurrent selection and RRGS: reciprocal recurrent genomic selection), individuals genotyped to calibrate the GS model (RRGS_HYB: genotyping parents and 1700 hybrids, RRGS_PAR: genotyping only parents), number of candidates (120 and 300) and the progeny test frequency (GGGG: every generation, GMGM: every two generations and GMMM: every four generations). The filled dots represent the means, calculated over 45 values

This advantage of the RRGS_HYB1700 scheme did not come without risks, and the higher gains also came with a larger variation in response compared to the less performing alternatives (Fig. 4). The coefficient of variation (CV) for the annual selection response of that best scheme reached 0.27, which was 35.6 % higher than that of RRS, and 19.1 % higher than the average CV over all the breeding schemes. The three following breeding schemes in the ranking of gain had similar levels of performance and CV for the annual response: RRGS_HYB1700 with progeny tests performed every four generations and 120 candidates (annual response of 0.39 % ± 0.23, +47.7 % compared to RRS), and RRGS_PAR with 300 candidates and calibration every four or two generations, which gave the same results (annual response of 0.38 % ± 0.22, +45.3 % compared to RRS).

### Inbreeding

**Fig. 5:**
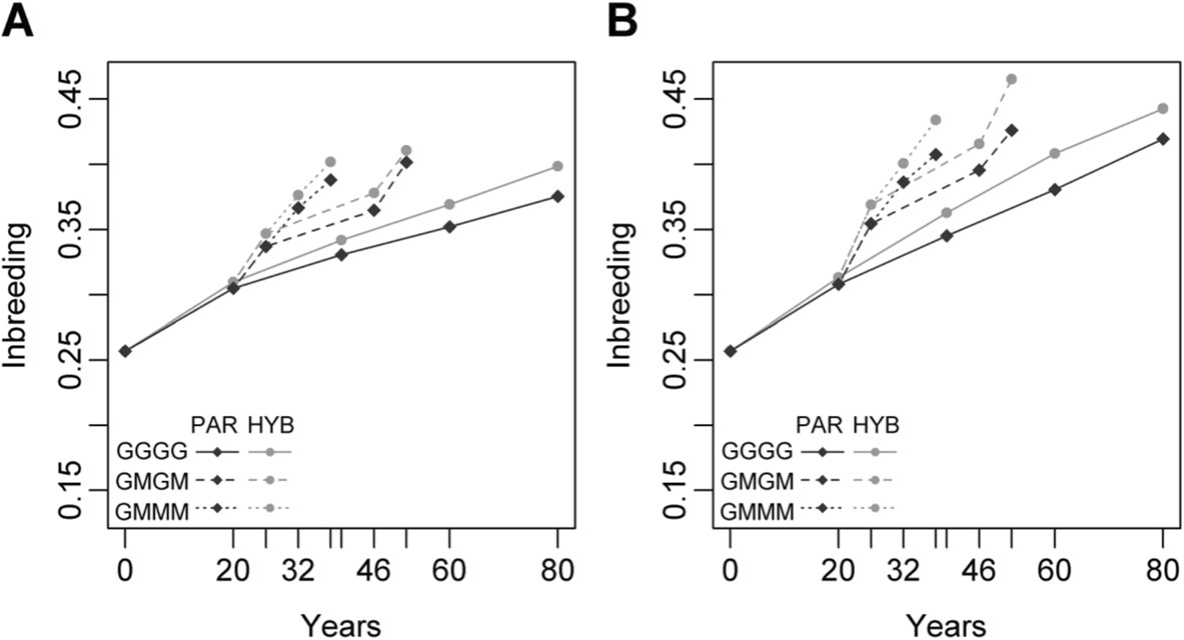
Inbreeding according to years and the reciprocal recurrent genomic selection (RRGS) breeding scheme in the Deli population using (**a**) 120 and (**b**) 300 candidates. The breeding scheme includes individuals genotyped to calibrate the GS model (parents and 1700 hybrids in RRGS_HYB, and only parents in RRGS_PAR) and progeny test frequency (GGGG: every generation, GMGM: every two generations and GMMM: every four generations). Means are calculated over 45 values

**Fig. 6:**
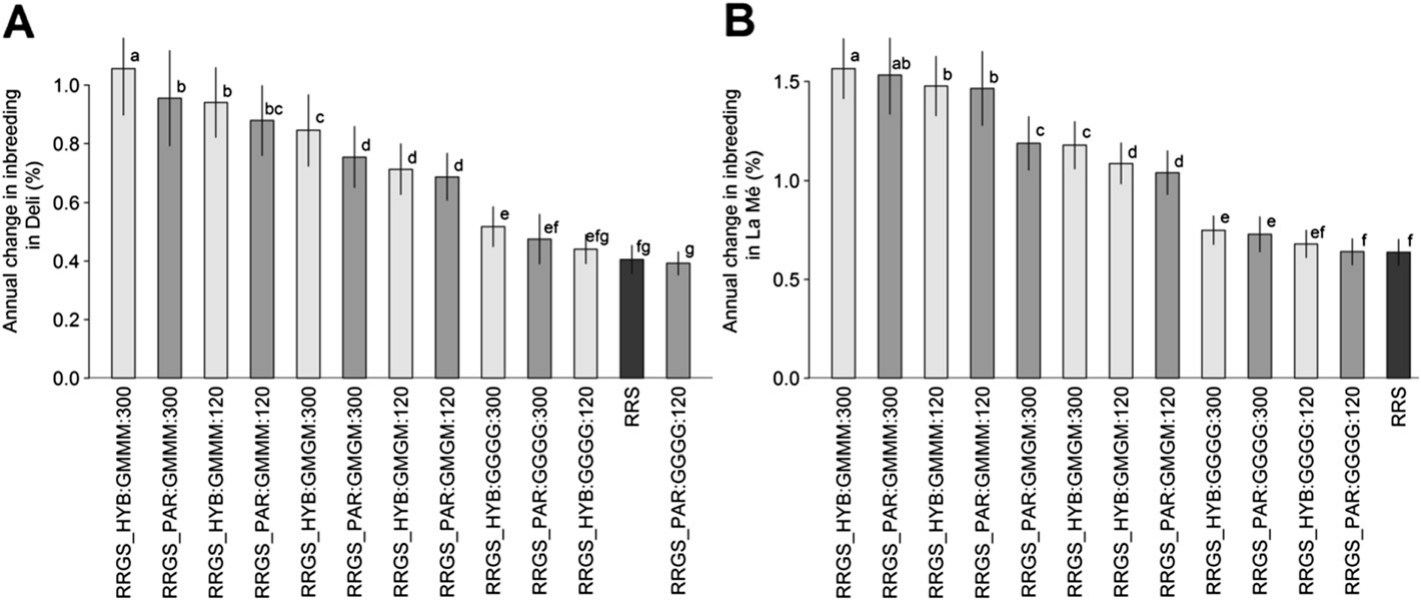
Ranking of breeding schemes according to their mean annual increase in inbreeding for (**a**) Deli and (**b**) La Mé populations. Inbreeding is expressed as a percentage of inbreeding in the parental populations in the initial generation (generation 0) per year. The breeding scheme includes the breeding strategy (RRS: reciprocal recurrent selection [black], RRGS: reciprocal recurrent genomic selection), individuals genotyped to calibrate the GS model (_PAR: genotyping only parents of progeny tests when calibrating the GS model [dark gray], _HYB: genotyping in addition hybrid individuals [light gray]), number of candidate individuals per population and generation (120 and 300; in RRS, the set of candidate individuals is limited to the 120 progeny-tested individuals of each parental population), the progeny test frequency (GGGG: every generation, GMGM: every two generations and GMMM: every four generations) and the number of genotyped individuals (0, 300, 1000 and 1700). Values are means over 45 replicates (3 numbers of QTL × 3 percentage of pleiotropic QTL × 5 replicates). Values with the same letter are not significantly different at *P* = 0.001. Bars indicate standard deviations

As expected, inbreeding increased with the generation turnover (see Fig. 5, with the example of RRGS in the Deli population). The annual increase in inbreeding (*∆F*_*y*_) with RRS was 0.41 % in Deli and 0.64 % in La Mé (expressed in percentage inbreeding in the initial parental populations) (Fig. 6). The factors affecting cumulative *∆F* (*∆F*_*c*_) over four generations and *∆F*_*y*_ were the same in both populations. *∆F*_*y*_ was mostly affected by the progeny test frequency, the number of candidates and the breeding strategy (*P* < 0.001). A decrease in progeny test frequency reduced the number of years per selection cycle, and therefore could substantially inflate *∆F*_*y*_. The number of candidates affected both *∆F*_*c*_ and *∆F*_*y*_. In Deli, *∆F*_*y*_ reached 0.77 % with 300 candidates versus 0.64 % with 120, and in La Mé it reached 1.16 % with 300 candidates versus 1.0 % with 120 (all differences significant at *P* < 0.001). Increasing the selection intensity by increasing the number of candidates from 120 to 300 therefore resulted in a subsequent increase in *∆F*_*y*_, due mainly to an increase in the co-selection of related candidates. With 120 candidates, they all belonged to different full-sib families due to the method used to mate the selected individuals. However, the sets of 300 candidates mostly consisted of pairs of full-sibs, which increased the probability of having full-sib individuals among the selected individuals. In addition, we noticed that RRGS_HYB was associated with a slightly higher *∆F*_*c*_ and *∆F*_*y*_ than RRGS_PAR. In Deli, RRGS_HYB led to a *∆F*_*y*_ of 0.75 % compared to 0.69 % with RRGS_PAR (*P* < 0.001). In La Mé, *∆F*_*y*_ was also higher with RRGS_HYB (1.12 %) than with RRGS_PAR (1.10 %), but this was not significant.

Finally, the four best breeding schemes previously identified in terms of annual selection response also had a high *∆F*_*y*_, with the exception of RRGS_PAR with progeny tests performed every two generations and 300 candidates, because of its higher progeny test frequency.

### Genetic correlation between BW and BN

**Fig. 7:**
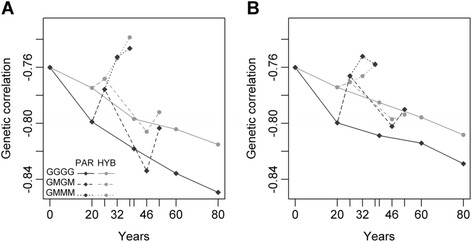
Genetic correlation between BN and BW in the Deli population according to years and the reciprocal recurrent genomic selection (RRGS) breeding scheme with (**a**) 120 selection candidates and (**b**) 300 candidates. The breeding scheme includes individuals genotyped to calibrate the GS model (parents and 1700 hybrids in RRGS_HYB and only parents in RRGS_PAR), the number of candidate individuals (120 and 300) and the progeny test frequency (GGGG: every generation, GMGM: every two generations and GMMM: every four generations). Means are calculated over 45 values

The evolution of the genetic correlation between BN and BW was similar for the Deli and La Mé populations. The magnitude of the genetic correlation between BW and BN increased markedly in the generations in which progeny tests were conducted (Fig. 7), while it usually decreased in the generations without progeny tests. In absolute value, the increase in the generations with progeny tests was greater than the decrease in the generations without progeny tests, so the correlation thus tended to increase over the four generations, except in the case where progeny tests were only performed in the first generation.

## Discussion

We showed that reciprocal recurrent genomic selection (RRGS) was a valuable method to achieve a long-term increase in the performance for a trait showing heterosis due to the multiplicative interaction between additive and negatively correlated components.

In our oil palm case study, RRGS was superior to traditional RRS as it allowed accurate selection of individuals without progeny tests. It led to a significant increase in the annual selection response, through generations of selection based on markers alone and, to a lesser extent, through an increase in the selection intensity. This advantage of RRGS over RRS was a consequence of the tradeoffs between the selection intensity (*i*), generation interval (*L*) and selection accuracy (*r*_*AÂ*_). RRGS could substantially increase the *r*_*AÂ*_ *× i / L* ratio, with the best breeding scheme (when considering only the annual selection response) being RRGS_HYB1700 with calibration every four generations and 300 candidates per generation and population. In our oil palm example, the annual selection response of this RRGS best strategy reached 0.45 %, compared to 0.26 % in RRS. Interestingly, both RRGS strategies with and without hybrid genotyping outperformed RRS. In RRGS_HYB, there was a marked increase in annual response when the number of genotyped individuals increased from 300 to 1000, but it was minor from 1000 to 1700 (although significant). Therefore, it seemed that genotyping more individuals would have been useless here. However, the number of hybrids to genotype should be dependent on the heterozygosity in the parental populations. Indeed, RRGS_HYB could perform better than RRGS_PAR because it exploited the within-cross phenotypic variation by associating it with the within-cross segregation of marker alleles. With a higher level of heterozygosity in the parental population, the phenotypic variation within crosses would likely increase, making more relevant hybrid genotyping of hybrids in order to capture this variation. On the contrary, in the extreme case of fully inbred and/or related parents, genotyping hybrids would become useless due to the absence of valuable within-cross variation.

Aspects other than just the expected annual selection response need to be taken into account when choosing an optimal breeding scheme. The RRGS_HYB1700 breeding scheme achieved the highest annual selection response but this also led to the highest variability in annual response (indicating a higher risk regarding the true genetic progress that could be achieved), and also the highest increase in inbreeding per year. Furthermore, the efficiency cost and operational complexity must be considered. RRGS_HYB would be more difficult and costly to implement than RRGS_PAR, because it would require the collection of more samples than RRGS_PAR, more genotyping and the gametes contributing to each hybrid individual would have to be inferred in order to identify the parental population of origin of marker alleles at each locus [6], which was assumed to be known in the simulation. Other breeding schemes could thus offer interesting alternatives, with good compromises between costs, operational complexity, expected annual selection response, risk regarding this expectation and evolution of inbreeding. Here the most interesting alternative was RRGS_PAR with 300 candidates and progeny tests every two generations. Indeed, it was among the best four breeding schemes in terms of annual selection response (0.38 %), but it also had a lower risk regarding its expected response and an inbreeding increase that was intermediate among all the scenarios studied, together with low cost and less operational complexity associated with the RRGS_PAR strategy. Furthermore, this RRGS_PAR scenario gives an opportunity to pool data from two progeny tests when making the calibration in the third generation, which would likely increase the accuracy in the two last generations and therefore the mean annual selection response over the four cycles.

The relative importance of the decrease in generation interval and the increase in selection intensity depends on the characteristics of the species. In oil palm, the length of the generation interval (20 years) is mostly due to the progeny tests, while sexual maturity is reached relatively early (within 3–4 years). This makes the species an excellent candidate for the implementation of early genomic evaluation, with RRGS having a high potential compared to RRS. By contrast, oil palm breeding populations have a quite narrow genetic base, with effective sizes under 10 [27], and this creates relatively small additive variances, therefore reducing the interest of increasing the number of candidates.

Our results confirmed the usefulness of GS for oil palm, in line with the simulation results of Wong and Bernardo [14]. However, we extended their results to a more general situation, closer to actual oil palm breeding program conditions, by applying GS to complex breeding populations and by considering two antagonistic traits, i.e. BN and ABW, which are crucial in oil palm breeding. Our results were consistent with the findings of our previous empirical study [12], which was conducted using an approach similar to the RRGS_PAR method used here. The accuracies we obtained in this previous study when applying GS to full-sibs of the training individuals could be compared with the accuracies we obtained here on the 180 full-sibs of the 120 progeny-tested individuals. We previously obtained mean accuracies of 0.74 for BN and BW using 105 Deli individuals to calibrate the GS model, which were the same values as in our present simulation. For the La Mé population, we previously obtained accuracies of 0.60 for BN and 0.65 for ABW with 74 individuals to calibrate the model (unpublished results), which in this case was smaller than the accuracies obtained here (0.75 for BN and 0.74 for BW). This was likely due to the fact that our previous training population was smaller than that used here (120). The consistency between the empirical results and our present simulations suggests that the actual genetic architecture for BN and BW could be close to the average scenario of our simulations, i.e. 500 QTL and 75 % of pleiotropic QTL.

Surprisingly, RRGS_HYB with 300 hybrid individuals genotyped had poor results, with a selection response lower than that of RRGS_PAR. We expected that RRGS_HYB would outperform RRGS_PAR even with a small number of genotyped hybrids due to the extra molecular information provided. We believe that RRGS_HYB300 performed poorly because, with such a small number of genotyped hybrids, the molecular relationships between the genotyped hybrids and their Deli and La Mé parents (or among genotyped hybrids) were biased and not compatible with the genealogical relationships involving their non-genotyped sibs. This could be due to the fact that the molecular relationships in RRGS_HYB were gametic relationships, where only half of the molecular data of hybrid individuals were used to relate them to each of their two parents. This could possibly be resolved by using more markers or by improving the computation of the ***H*** matrix used in RRGS_HYB, which requires further investigation.

Actually, we chose different GS models for RRGS_HYB and RRGS_PAR for computational reasons: two ***G*** matrices were required in the model chosen for RRGS_PAR. When no molecular data from hybrids were used, the ***G*** matrices were small (from 120 × 120 to 420 × 420). However, using the same model for RRGS_HYB would mean having two large ***H*** matrices (from 13,740 × 13,740 to 14,340 × 14,340). This would result in a high computation time that would be hard to manage in a simulation study due to the many replicates, and memory problems. For this reason, we used a different GS model for RRGS_HYB, requiring a single relationship matrix. Hence, in RRGS_HYB, the breeding values of the Deli and La Mé parents and hybrid individuals were assumed to belong to the same distribution with a common additive variance, which was out of line with the actual situation. However, this should not be a problem here as the mixed models were used to predict GEBV, not to estimate genetic parameters.

### Management of genetic variability

We found that the increase in inbreeding (*ΔF*) per generation was higher with GS than with RRS, which was somewhat out of line with previous animal breeding results, as reviewed in Bouquet and Juga [28], where GS was found to reduce *ΔF* per generation compared to traditional breeding. According to these authors, this occurred because the Mendelian sampling terms (i.e. individual genetic effects) were more accurately estimated with GS than with phenotypic selection, thus reducing the probability of selecting sibs and consequently the *ΔF* per generation. We assumed the different trend existing in our study was related to the drop in accuracy observed for non-progeny-tested individuals, which occurred because the calibration of the GS model was based on the progeny tests of only 120 individuals per population. Consequently, our estimates of Mendelian sampling terms were likely not as accurate as those obtained in animal species. However, it was not clear why RRGS_HYB led to a higher increase in *ΔF* than noted with RRGS_PAR, whereas it was more accurate. Depending on the reduction in generation interval allowed by GS (which is dependent on the species), Bouquet and Juga [28] noted that this would lead to a lower or similar annual *ΔF* than in traditional breeding. In oil palm, the reduction in the generation interval allowed by GS is very high (only 38 years to complete four cycles when calibration was carried out only in the first generation, compared to 80 years for traditional RRS). Consequently, the best breeding schemes in terms of annual response had the highest annual increase in inbreeding, with the exception of RRGS_PAR with 300 candidates and progeny tests performed every two generations, which had an intermediate annual *ΔF*.

Good genetic variability management is necessary to avoid inbreeding depression in parental populations, which has been reported in oil palm [10, 29, 30], and to maintain the genetic progress potential over the long term. Furthermore, the negatively correlated BN and BW studied in this simulation are key traits for oil palm breeding, and when dealing with antagonistic traits breeders must find a compromise between the selection response, variance in the response induced by antagonistic traits and *ΔF* [31]. Therefore, the RRGS breeding schemes we presented here should be combined with methods to explicitly manage genetic diversity and inbreeding. The simplest inbreeding management method is to increase the number of selected individuals, which would slow down the increase in inbreeding, possibly with only a small reduction in selection response [28]. Another option that does not necessarily lead to gain losses is optimal contribution selection [32] and its extension in the GS context [33]. This involves the use of the genetic value of individuals and their relationships with other individuals to determine their contribution to the following generation, in order to maximize genetic gain at a desired inbreeding rate under the assumption of random mating among selections. A step further is mate selection [34, 35], where the optimum contribution is applied to mates among all candidates, so that selection and mating are simultaneously handled for improved management of inbreeding beyond what is expected by random mating. Mate selection optimizes the number of parents to be selected, the actual matings between them and the distribution of the contribution in the offspring of these mates, in order to maximize the expected selection response in the following generation while respecting a restriction on the expected increase in inbreeding.

### Genomic selection model

Here we studied a GS approach to select individuals within two parental populations for their crossbred performance, as in several animal studies [6, 36–38] and in maize [39]. We used models with population-specific effects of SNP alleles, using either a parental model with two independent parental effects (RRGS_PAR) or by distinguishing alleles depending on their population of origin (RRGS_HYB). However, Ibánez-Escriche et al. [36] and Toosi et al. [37] suggested that GS models using crossbred populations to predict GEBV of parental pure breeds may not need to fit breed-specific SNP effects, especially with high marker density. However, we did not considered this point, as in Ibánez-Escriche et al. [36] breed-specific allele models performed better than models with allele effects common to all breeds when the breeds where distantly related, which was the case in our simulations.

In this study we considered that heterosis in bunch production was a consequence of the multiplicative interaction between the negatively correlated bunch number and bunch weight, both assumed here to have complete additive genetic determinism. This multiplicative interaction between complementary component differences in the parents is a heterosis model without dominance, but heterosis in a multiplicative trait can also be due to the multiplicative interaction of component dominance [7]. In this case, dominance in the component traits generates heterosis in the complex trait, to a greater extent than the dominance in the components, due to the multiplicative nature of the complex trait. Here we did not study the effect of this type of genetic determinism (or a combination of the two types). This would require further investigation, which could be done by modifying the script used for our simulations and including dominance effects in the GS models (see for instance Su et al. [40] for a GBLUP model including dominance effects).

### Genetic correlation between BW and BN

Wu and Sánchez [41] showed that in a model associating pleiotropic and non-pleiotropic QTL, simultaneous selection on the two traits increased the magnitude of the genetic correlation. Presumably their result applied in the case where selection was highly accurate, such as when based on progeny tests, which was not the case here when selection was made on markers alone using a GS model calibrated with a small training set.

## Conclusions

Reciprocal recurrent genomic selection (RRGS) appeared as a valuable method to achieve a long-term increase in the performance for a trait showing heterosis due to the multiplicative interaction between additive and negatively correlated components. In our oil palm case study, RRGS was superior to traditional RRS in terms of annual selection response as it could decrease the generation interval and increase the selection intensity. With 1700 genotyped hybrids, calibration every four generations and 300 candidates per generation and population, selection response of RRGS was 71.8 % higher than RRS. RRGS without genotyping hybrid individuals, with calibration every two generations and 300 candidates was a relevant alternative, as a good compromise between the annual response, risk around the expected response, increased inbreeding and cost.

## Abbreviations

BN: Bunch number
BW: Bunch weight
(G) EBV: (Genomic) estimated breeding value
GS: Genomic selection
RRGS: Reciprocal recurrent genomic selection (_HYB: model calibration using parental and hybrid genotypes, _PAR: model calibration using only parental genotypes)
RRS: Reciprocal recurrent selection
QTL: Quantitative trait loci
